# Intracranial mapping of linguistic structure building during listening speech comprehension

**DOI:** 10.1162/IMAG.a.1158

**Published:** 2026-03-04

**Authors:** Lingxi Lu, Lu Luo, Siqi Li, Na Xu, Xiongfei Wang, Guoming Luan, Qian Wang, Jia-Hong Gao

**Affiliations:** Cognitive Science and Allied Health School, Institute of Life and Health Sciences, Beijing Language and Culture University, Beijing, China; Key Laboratory of the Cognitive Science of Language (Ministry of Education), Beijing, China; School of Psychology, Beijing Sport University, Beijing, China; Center for Motor Control and Disease, Key Laboratory of Brain Functional Genomics, East China Normal University, Shanghai, China; Division of Brain Sciences, Changping Laboratory, Beijing, China; Beijing Key Laboratory of Epilepsy, Epilepsy Center, Sanbo Brain Hospital, Capital Medical University, Beijing, China; School of Psychological and Cognitive Science, Peking University, Beijing, China; State Key Laboratory of General Artificial Intelligence, Peking University, Beijing, China; IDG/McGovern Institute for Brain Research, Peking University, Beijing, China; Center for the MRI Research, Peking University, Beijing, China; Beijing City Key Lab for Medical Physics and Engineering, School of Physics, Peking University, Beijing, China; National Biomedical Imaging Center, Peking University, Beijing, China

**Keywords:** speech comprehension, linguistic structure, intracranial EEG, frequency tagging

## Abstract

Understanding how the human brain processes linguistic structures during speech comprehension is essential for understanding the neural mechanisms of language processing. Despite prior efforts to map the cortical organization of hierarchical linguistic structures, a comprehensive characterization with both high spatial and temporal resolution remains limited. To address this issue, our study applied intracranial stereo-electroencephalography (sEEG) recordings to map the neural tracking at distinct linguistic timescales in the human brain in 20 participants. We employed a frequency tagging paradigm to tag syllable-, phrase-, and sentence-level structures at specific frequencies. The findings revealed robust neural tracking in the fronto-temporal regions at different linguistic levels, with the primary auditory cortex (Heschl’s gyrus) exhibiting the strongest activity at the syllabic level, and the superior temporal and inferior frontal regions engaged in the building of higher-level phrasal and sentential structures. Importantly, the neural tracking responses to hierarchical linguistic structures were spatially differentiated across different brain regions. Furthermore, high-gamma responses detected by intracranial electrodes within the left language network were observed to be sparsely distributed, predominantly concentrated in specific fronto-temporal sites. These results suggest spatially distributed functional specialization in the brain for tracking different levels of linguistic structure, as reflected in distinct neural oscillatory responses. This study advances our understanding of the functional organization in cortical regions during auditory speech comprehension and may offer insights for aligning brain processes in language models.

## 1 Introduction

An essential cognitive process in human brain during language comprehension is the efficient analysis and integration of a hierarchy of linguistic structures, including syllables, words, phrases, and sentences (Berwick et al., 2013; Chomsky, 1957). Exploring how the brain rapidly processes speech linguistic structures across different temporal scales, ranging from syllables lasting hundreds of milliseconds to sentences lasting seconds, is critical for understanding the neural mechanisms underpinning language comprehension. Recording techniques with high temporal resolution, such as magnetoencephalography (MEG) and electroencephalography (EEG), have been widely used to address this issue. It has been shown that low-frequency neural oscillations in the delta and theta bands, aligning with phrasal and syllabic speech rates, play a critical role in processing intelligible speech (Ding & Simon, 2013; Doelling et al., 2014; Lizarazu et al., 2021; Luo & Poeppel, 2007; Molinaro & Lizarazu, 2018).

An inspiring work by Ding et al. (2016) employed a frequency-tagging paradigm in their MEG recording to identify low-frequency neural track responses to hierarchical linguistic structures. They designed connected speech stimuli with 250-ms syllables and manipulated the linguistic hierarchy by tagging two-syllable phrases at 500 ms and two-phrase sentences at 1 s. This research successfully captured neural tracking responses entrained to linguistic structure building, revealing distinct spectral peaks at 4 Hz for the syllabic rate, at 2 Hz for the phrasal rate, and at 1 Hz for sentential rate (Ding et al., 2016). Following this, recent studies using frequency-tagged M/EEG have provided valuable insights into the modulation of low-frequency cortical tracking to speech structures by different cognitive factors, such as attention and language experience (Ding et al., 2018; Har-shai Yahav & Zion Golumbic, 2021; Lu et al., 2023; Reetzke et al., 2021), as well as by different brain states, including consciousness and sleeping (Gui et al., 2020; Makov et al., 2017).

Despite the excellent temporal resolution provided by non-invasive M/EEG techniques, it is still challenging to localize the specific brain networks engaged in concurrent neural tracking to speech structures in high spatial resolution. MEG source estimation provides an initial glimpse into the localization of neural clusters involved in this process. For example, Sheng et al. (2019) applied a novel MEG source estimation algorithm to map the neural activations at tagged frequencies corresponding to the syllabic, phrasal, and sentential rates, revealing the involvement of the superior temporal gyrus in processing speech structures across all three levels, with additional recruitment of the anterior temporal lobe and left inferior frontal gyrus selectively for processing phrases and sentences (Sheng et al., 2019). Activation in the left inferior frontal and posterior parietal regions was observed during the representation of phrase structures, as revealed by MEG source estimation (Har-shai Yahav & Zion Golumbic, 2021). These brain regions partially overlapped with the neural networks identified by the intracranial recordings in Ding et al. (2016), which highlighted the involvement of the bilateral superior temporal gyrus and left inferior frontal gyrus in processing phrase or sentence structures. These findings are further complemented by recent iEEG evidence showing that linguistic processing follows a joint and hierarchical organization in the auditory cortex (Keshishian et al., 2023). It is worth noting that Ding et al. (2016) documented both MEG in healthy adults and intracranial electrocorticography (ECoG) in subjects’ cortical surface, yet ECoG did not allow for local recording in deeper neural clusters, such as the Heschl’s gyrus (HG), which is essential for encoding acoustic features in speech. Intracranial stereo-electroencephalography (sEEG) provides high spatial and temporal resolutions, as well as depth recordings to address aforementioned challenges.

Moreover, intracranial EEG allows for reliable recordings of high-gamma neural activities, which are closely linked to neuronal firing rate (Ray & Maunsell, 2011). Research has indicated that the superior temporal and inferior frontal region in the left fronto-temporal language network exhibit increased high-gamma activities during the merge operation of visual words into phrases (Nelson et al., 2017) and sentence processing (Woolnough et al., 2023). The enhancement of high-gamma activity related to auditory language processing provides additional useful information to localize indispensable language regions (Aron et al., 2021; Kojima et al., 2013). Hence, intracranial EEG offers an opportunity to compare high-gamma neural activities with low-frequency neural tracking that was typically examined in M/EEG studies, allowing for a thorough understanding of the neural networks underpinning linguistics structure building during speech comprehension (Aron et al., 2021).

In this study, building on the frequency-tagging paradigm developed by Ding et al. (2016), we extend this approach by using depth sEEG recordings to achieve fine-grained spatial resolution, particularly in deeper cortical regions such as HG, and by examining both low-frequency tracking and high-gamma responses. In our frequency-tagging framework, hierarchical linguistic structure is operationalized as distinct temporal rates (syllables at 4 Hz, phrases at 2 Hz, and sentences at 1 Hz) so that each linguistic level corresponds to a fixed rhythmic timescale embedded in the speech input. Our focus is on the neural tracking responses in the auditory cortex, including HG and superior temporal region, as well as the left fronto-temporal language network, including the inferior frontal gyrus and middle temporal region. By analyzing low-frequency and high-gamma brain activities during continuous speech processing, we aim to present a precisely-localized neural mapping of linguistic structures during speech listening comprehension.

## 2 Materials and Methods

### 2.1 Participants

Twenty subjects (7 females and 13 males, with a mean age of 28.3 ± 8.3 years) who were undergoing clinical assessment for epilepsy treatment in Beijing Sanbo Brain Hospital participated in this study. All the participants were Mandarin Chinese speakers who were right-handed and had normal hearing abilities as indicated in their clinical evaluations. Prior to their participation, written informed consent was obtained from all participants, or from the parents of minors. The experimental protocol received approval from the Ethics Committee of the Sanbo Brain Hospital, Capital Medical University. The implantation of electrodes in the subjects’ brains was carried out solely in accordance with clinical requirements. Eleven subjects were implanted with left hemisphere electrodes, three were implanted with right hemisphere electrodes, and six were implanted with electrodes in both hemispheres. The demographic information and implantation details for each subject are summarized in Table 1.

**Table 1.** Demographic and implantation details for participants.

Subj. ID	Age	Gender	Dominant hand	Hemisphere of implantation	Number of contacts
1	34	Male	Right	Left, right	84
2	28	Male	Right	Left	93
3	46	Female	Right	Left, right	88
4	22	Male	Right	Left	105
5	44	Female	Right	Left, right	61
6	19	Female	Right	Left, right	46
7	29	Female	Right	Left	67
8	16	Male	Right	Left	88
9	19	Male	Right	Right	98
10	21	Male	Right	Right	96
11	24	Male	Right	Left, right	125
12	35	Male	Right	Left	87
13	23	Female	Right	Left, right	101
14	32	Male	Right	Left	82
15	22	Male	Right	Left	85
16	28	Male	Right	Left	100
17	29	Male	Right	Left	80
18	28	Female	Right	Left	108
19	27	Male	Right	Right	83
20	40	Female	Right	Left	66

### 2.2 Stimuli

Three types of 10-s speech sequences were prepared, which included syllables, two-syllable phrases, and two-phrase sentences. For illustration, example stimuli included the syllables “万均齐见”, the phrases “游客英雄”, and the sentence “朋友请客”. Each syllable in the speech sequences lasted for 250 ms, with no inter-syllable gaps to eliminate any influence of speech rate or other prosodic cues on structural processing. This led to a syllable-level presentation frequency tagged at 4 Hz. Phrases, consisting of two syllables, were presented at a rate of 2 Hz. Sentences, composed of a noun phrase and a verb phrase, contributed an additional presentation frequency of 1 Hz at the sentential level.

Forty 10-s sentence sequences were created by randomly selecting 10 sentences from two sets of 50 two-phrase sentences each. Specifically, 20 sentence sequences were generated from sentence pool A, and the other 20 from sentence pool B. These two-phrase sentences were adapted from a prior study by Sheng et al. (2019) (refer to Supplementary Materials for details). Subsequently, by randomly selecting ten noun phrases and ten verb phrases from two pools of 50 sentences each, a total of 20 sequences of noun phrases and 20 sequences of verb phrases were generated. Finally, 40 syllable sequences were generated by shuffling the order of syllables in the sentence sequences.

### 2.3 Procedures and apparatus

The current experiment included three conditions (syllables, phrases, sentences), each consisting of 40 trials (40 speech sequences). To accommodate the clinical setting, the order of 40 trials for the syllable and sentence conditions was randomized and split into 4 sessions of 10 trials, allowing subjects to take breaks if needed. The 40 trials for the phrase condition were divided into 20 noun phrase trials and 20 verb phrase trials. The 20 trials of noun or verb phrase sequences were randomized and split into 2 sessions of 10 trials. The order of presentation order for the three conditions was counterbalanced using a Latin square design across subjects. For the phrase condition, the presentation order of noun or verb phrases was balanced across subjects. In each trial, participants were presented with a 10-s speech stimulus and instructed to attentively listen to the speech. Within each trial, the 10-s speech stimulus was presented continuously without additional silent gaps between syllables, phrases, or sentences. Between trials, a silent random interval of 1-2 s was inserted. The experimenter initiated each session by pressing a button after receiving confirmation from the subjects that they were ready to proceed.

The experiment was carried out in the ward beside the subject’s hospital bed. Subjects were sitting on their bed before a mobile bedside table. Speech materials were sampled at 22.05 kHz, and presented binaurally to participants via a laptop using Adobe Audition. Sounds were delivered with a Creative Sound-Blaster X-Fi sound card (Creative Technology Ltd, Singapore) and Etymotic Research (ER-3A) insert earphones. The sound pressure level was adjusted to a comfortable level and maintained consistently across subjects. The entire duration of the experiment was approximately 30 min. Due to clinical constraints, the patients were not formally tested for language lateralization.

### 2.4 SEEG recordings and localization

SEEG signals were recorded when participants were listening to the speech materials. The intracranial electrode with a diameter of 0.8 mm consists of 12 to 18 independent recording contacts, each 2 mm long and spaced 1.5 mm apart from one another. The reference site was attached to the skin located on the subjects’ foreheads. The neural responses were collected using the Nicolet clinical amplifier at a sampling rate of 2048 Hz for 1 subject (subj.ID 5) and at 512 Hz for the other 19 subjects.

**Fig. 1.**
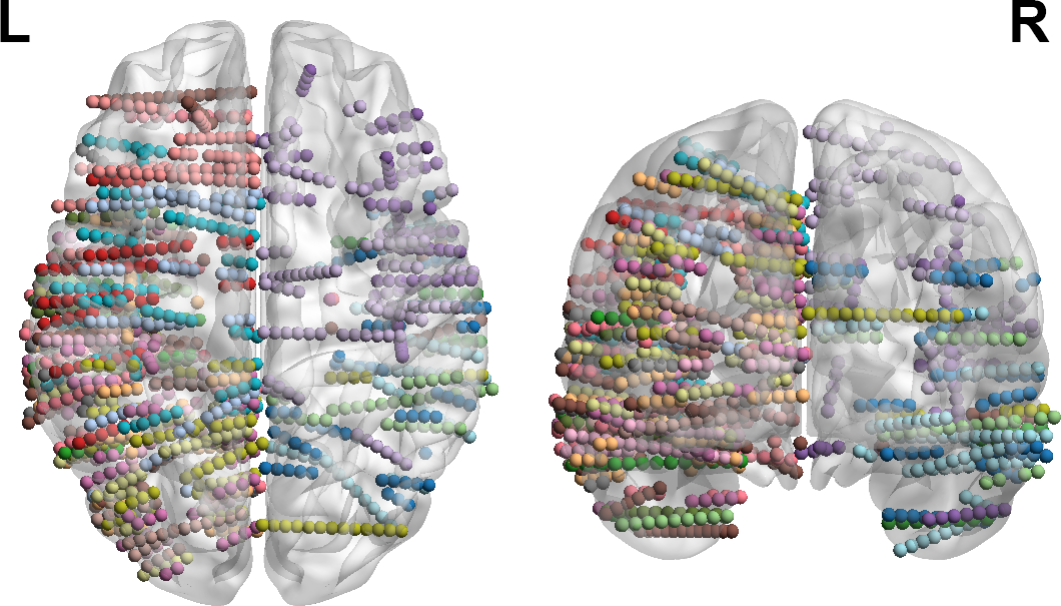
Coverage of recording contacts in 20 subjects. For all the 1743 contacts, 74.24% of them were localized in the left hemisphere, and 25.76% were in the right hemisphere. Each color represents contacts implanted in an individual subject.

Cortical reconstruction and segmentation were individually computed for each subject based on the pre-electrode implantation T1-weighted image using Freesurfer. Subsequently, the CT scan, along with the electrode localizations and anatomical images, underwent co-registration and normalization to the MNI-152 template. The automated anatomy labeling (AAL) atlas was used to label the brain region of the co-registered electrodes, and contacts outside the cortex space were excluded. The number of contacts included in analysis was summarized in Table 1. The contact locations were identified and visualized with MNI coordinates using the Brainstorm toolbox and the BrainNetViewer toolbox. The Brainstorm toolbox was also employed to render, smooth, and inflate cortical surfaces for visualization. Figure 1 displays the localization of 1743 analyzed contacts among 20 subjects in the MNI-152 space, with 1294 contacts in the left hemisphere and 448 contacts in the right hemisphere.

### 2.5 Data pre-processing

In analyzing low-frequency neural tracking responses (broadband spectrum), the raw data were epoched from -0.5 to 10 s relative to the onset of the 10-s speech sequence. After baseline (-0.5-0 s relative to the onset) removal, the first 1 s after the stimulus onset was excluded to avoid the influence of transit auditory responses. The remaining 9-s data were then subjected to fast Fourier transformation (FFT), yielding a frequency resolution of 0.11 Hz. The inter-trial phase coherence (ITPC) was then computed across all trials in the same condition for each contact separately as follows:

$$\text{ITPC}=\left|\frac{1}{n}\sum_{r=1}^{n}e^{i\varphi_{r}}\right|$$

where *n* represents the number of trials, and *φ*_*r*_ indicates the Fourier phase angle of the stimulus on trial *r*. Following that, the ITPC was normalized using Rayleigh’s Z transformation method (Cohen, 2014), yielding z-scored ITPC to reduce individual variability. The z-scored ITPC between 0.5 and 4.5 Hz were extracted to examine the low-frequency neural tracking responses at the syllable-level rate of 4 Hz, the phrase-level rate of 2 Hz, and the sentence-level rate of 1 Hz. To test for significant tracking, z-scored ITPC values were compared against a threshold derived from the null hypothesis that, in the absence of phase locking, only baseline-level responses would be observed.

In analyzing high-gamma responses (high-gamma envelope spectrum), the raw data at each contact were first bandpass filtered at 60–100 Hz, followed by the extraction of the instantaneous amplitude envelope by applying Hilbert transformation. The resulting high-gamma envelope data were converted to the frequency domain using the FFT, and the ITPC response was calculated and z-score normalized evaluate the high-gamma neural tracking responses of speech linguistic structures. The choice of the 60–100 Hz band was constrained by the online filters of the clinical recording system, which limited the upper usable frequency to 100 Hz. This high-gamma range has also been used in previous intracranial studies operating under similar recording constraints (Lu et al., 2019).

### 2.6 Statistical analysis

We applied linear mixed-effects models (LMMs) on z-scored ITPC to examine how tracking responses varied across linguistic levels (syllable, phrase, sentence), hemisphere (left, right), and regions of interest (ROIs) within the fronto-temporal network, including HG, superior temporal gyrus (STG), middle temporal gyrus (MTG), inferior temporal gyrus (ITG), and inferior frontal gyrus (IFG), as defined by the AAL atlas. In the full model, linguistic level, hemisphere, and brain region were included as fixed factors along with all interaction terms. Random intercepts were included for subject and recording contact to account for between-subject and between-contact variability inherent to intracranial recordings. Each recording contact represented a unique physical electrode contact implanted in a single participant; thus, contacts were fully nested within participants. Following identification of a significant three-way interaction, separate two-way LMMs (linguistic level × brain region) were fitted for the left and right hemispheres to examine hemisphere-specific response patterns. Post-hoc pairwise contrasts were performed using estimated marginal means (emmeans), with false discovery rate (FDR) correction for multiple comparisons.

To test region- and linguistic-level differences in structure sensitivity, we implemented mixed-effects logistic regression models. Response selectivity was defined as a binary variable (*f*+/*f*-), indicating whether a given contact exhibited significant neural entrainment at a given linguistic level. Fixed effects included cortical region (ROI: HG, STG, MTG, ITG, IFG), hemisphere (left, right), and linguistic level (syllable, phrase, sentence), along with their interactions. Subject and contact were included as random intercepts. Statistical significance of fixed effects was assessed using likelihood ratio tests comparing nested models. Post-hoc comparisons were performed using estimated marginal means with false discovery rate (FDR) correction for multiple comparisons.

## 3 Results

### 3.1 Low-frequency neural tracking to speech linguistic structures

**Fig. 2.**
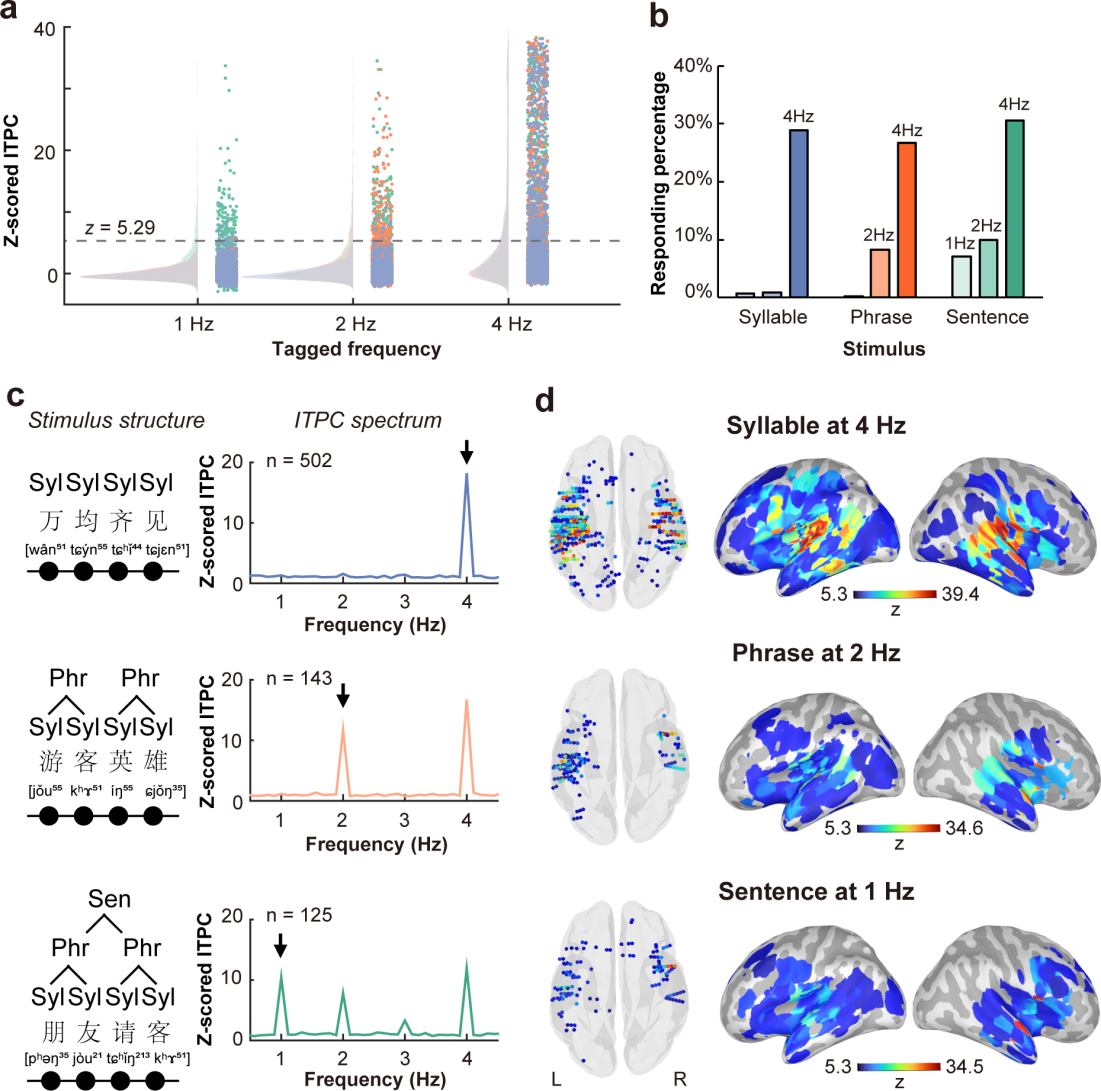
Low-frequency responses to speech linguistic structures under three conditions. (a) Peak responses at all contacts are shown. Blue dots represent the syllable condition, yellow dots represent the phrase condition, and green dots represent the sentence condition. The distributions of responses are displayed on the left side of each panel. The dashed line indicates the contact-level significance threshold (corresponding to *p* = 0.001, Bonferroni corrected). Contacts with responses exceeding this threshold were considered significant. (b) The responding percentage among all 1743 contacts was summarized, showing a high responding percentage of 28.80% at 4 Hz for the syllable condition, 26.68% for the phrase condition, and 30.64% for the sentence condition. Tracking responses at the phrase level were observed at 2 Hz for both the phrase (8.20%) and sentence (9.87%) conditions, while sentence-level tracking was detected at 1 Hz solely for the sentence condition (7.17%). (c) Averaged spectral responses at 4 Hz in the syllable condition, 2 Hz in the phrase condition, and 1 Hz in the sentence condition. Examples of the stimulus structures are shown with Chinese characters and their International Phonetic Alphabet (IPA) transcriptions. (d) Localization of responsive contacts and rendered activation mapping on the cortex surface for neural tracking at the syllabic, phrasal, and sentential rates.

A threshold criteria of z = 5.29 (corresponding to a *p* value of 0.001 in a two-tailed normalized distribution, adjusted by Bonferroni correction for multiple comparison in all electrodes and three linguistic conditions) was applied to the z-scored ITPC. The response distribution of all contacts that exceeded the threshold criteria for each condition is illustrated in Figure 2a.

Consistent with the prediction, after contact-level thresholding we observed a population of 502 contacts (responding percentage = 28.80%) that were responsive at 4 Hz when the stimuli were syllables, whereas less than 1% of the contacts responded at the other tagged frequencies (0.86% at 2 Hz and 0.69% at 1 Hz) in the syllable condition (Fig. 2b). In the phrase condition, 143 contacts (8.20%) responded at the phrasal rate of 2 Hz, and 465 contacts (26.68%) responded at the syllabic rate of 4 Hz. The responding percentage was 0.17% at 1 Hz in the phrase condition. When sentences were presented, 7.17%, 9.87%, and 30.64% of the contacts responded at 1 Hz, 2 Hz, and 4 Hz, respectively. The proportion of electrodes exceeding the threshold reflected the extent and distribution of responsive sites across the brain.

The averaged ITPC spectrum of responsive contacts in each condition (responsive at 4 Hz for syllables, at 2 Hz for phrases, and at 1 Hz for sentences) showed robust spectral peaks at tagged frequencies (Fig. 2c). Concurrent neural tracking to phrasal and sentential structure was observed when higher-order linguistic constructs were employed in continuous speech. Figure 2d illustrates the localization of responsive contacts and activation pattern on the cortex surface. There was pronounced activation in the temporal auditory region at a syllabic rate of 4 Hz, indicating strong low-frequency tracking of sound amplitude fluctuations at the acoustic level. Additionally, contacts showing phrasal- and sentential-rate tracking were distributed across both hemispheres, extending beyond primary auditory cortex.

LLM analysis confirmed that the pattern of tracking responses (Fig. 2d) across linguistic levels differed significantly across regions and between hemispheres, as indicated by a significant region × hemisphere × linguistic level interaction (F(8, 1062.0) = 2.997, *p* = 0.0025). Follow-up hemisphere-specific analyses further revealed significant region × linguistic level interactions in both hemispheres (left: F(8, 772.0) = 57.26, *p* < 0.001; right: F(8, 421.18) = 18.20, *p* < 0.001), indicating that hierarchical tracking patterns varied across cortical regions. Post-hoc comparisons showed that primary auditory cortex (HG) in both hemispheres exhibited the strongest entrainment at the syllabic frequency compared with phrasal and sentential rates (all FDR-corrected *p* < 0.001). In the left hemisphere, HG showed significantly higher ITPC than all other regions at the syllabic rate (all *p* < 0.001), whereas in the right hemisphere, both HG and STG displayed comparably strong syllabic tracking, exceeding all other regions (all corrected *p* < 0.001). The STG also exhibited a graded profile (syllable > phrase > sentence, all corrected *p* < 0.05), with the right STG displaying enhanced activation at the phrasal rate relative to other right-hemispheric regions (all corrected *p* < 0.01). Together, these results indicate a hierarchical organization of linguistic tracking, characterized by strong syllabic entrainment in the auditory cortex and progressive engagement of higher-order temporal and frontal regions at longer linguistic timescales. Statistical details of all pairwise post-hoc comparisons are provided in the Supplementary Materials (Supplementary Tables S1-S4, Supplementary Figs. S1-S2).

**Fig. 3.**
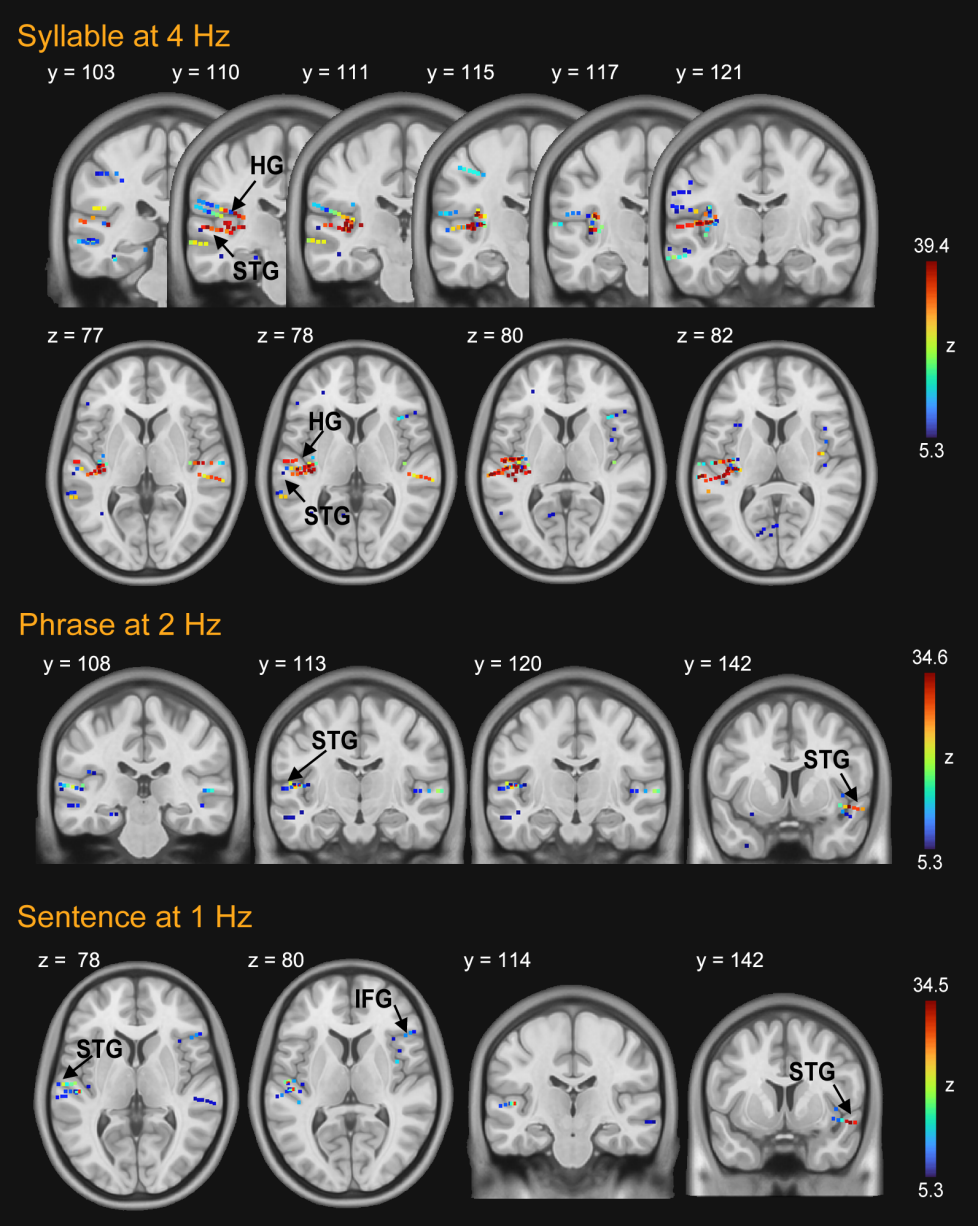
The localization of stereotactical contacts with significant low-frequency tracking. Strong responses at 4 Hz were noted in both the HG and STG. Strong responses were observed in the HG, with additional responsive contacts in the outer region of the STG. The STG participation was noted in phrasal rate tracking at 2 Hz, and a smaller number of contacts were detected during sentential rate tracking at 1 Hz, pinpointed in both the IFG and STG area. HG, Heschl’s gyrus; STG, superior temporal gyrus; IFG, inferior frontal gyrus.

As illustrated in Figure 3, the primary auditory cortex (HG) contained many of the contacts showing syllabic-rate (4 Hz) tracking, with additional responsive contacts observed in superior and inferior temporal regions. The high responding percentage of contacts in the HG region at 4 Hz (as shown in Table 2) suggests a strong involvement of the primary auditory cortex in tracking the low-level speech envelope. In the phrase condition at 2 Hz, a large number of responsive contacts were observed in the superior temporal lobe, with a higher proportion of contacts in this region than in the syllable condition. Furthermore, in the sentence condition at 1 Hz, the involvement of the IFG region was noted in the construction of sentential rate. Table 2 provides a detailed breakdown of the percentage of responsive sEEG contacts in the fronto-temporal brain regions, which play a fundamental role in supporting efficient language processing during speech comprehension.

**Table 2.** Responding percentage of sEEG contacts in the fronto-temporal region.

		Syllable			Phrase			Sentence		
Brain region	Contact number	1 Hz	2 Hz	4 Hz	1 Hz	2 Hz	4 Hz	1 Hz	2 Hz	4 Hz
Left										
HG	23	9%	17%	96%	0	26%	91%	13%	39%	100%
STG	90	0	6%	84%	0	44%	84%	31%	52%	86%
MTG	187	2%	0	32%	0	13%	37%	6%	12%	44%
ITG	68	0	0	16%	0	3%	25%	3%	4%	24%
IFG	40	0	0	28%	0	5%	10%	18%	18%	23%
Right										
HG	4	0	0	100%	0	50%	50%	0	75%	100%
STG	20	0	20%	85%	0	75%	65%	50%	55%	80%
MTG	63	0	0	33%	0	11%	22%	17%	8%	43%
ITG	53	0	0	47%	0	0	28%	2%	0	53%
IFG	23	0	0	22%	0	4%	26%	39%	9%	9%

HG, Heschl’s gyrus; STG, superior temporal gyrus; MTG, middle temporal gyrus; ITG, inferior temporal gyrus; IFG, inferior frontal gyrus.

**Fig. 4.**
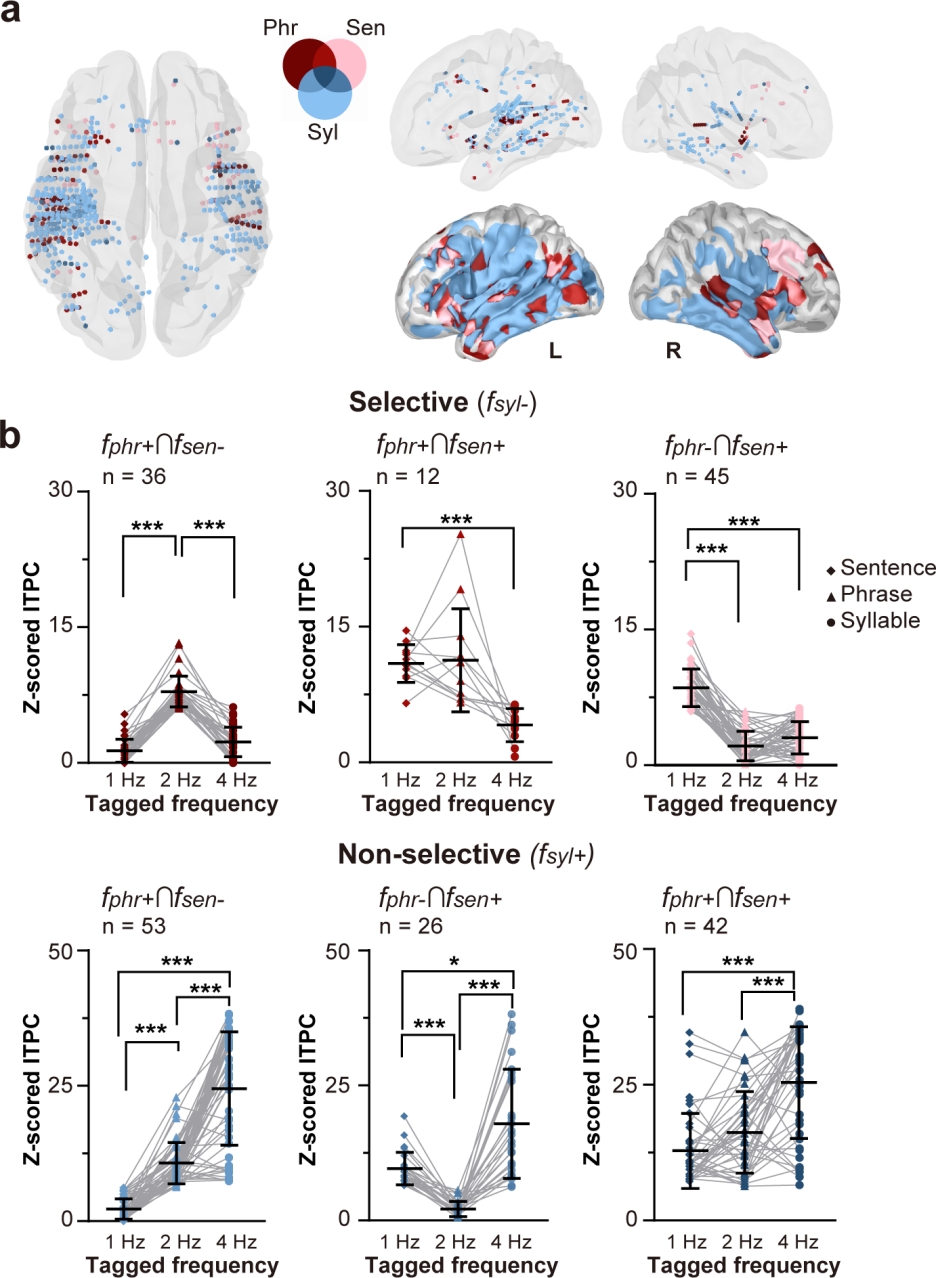
Spatially differentiated tracking of speech linguistic structures. (a) Some neural clusters (in red and pink) showed selective sensitivity to phrase and sentence structures but not syllables, primarily in the fronto-temporal region and temporo-parietal junction. (b) Distinct populations of neural clusters exhibited non-selective responses (in blue) to both higher-order structures and basic syllabic fluctuations, indicating a concurrent process of linguistic structures at different levels. Z-scored ITPC was compared among different linguistic levels by paired sample *t* tests with Bonferroni correction. Grey lines connect data from the same contact. Note that electrode coverage differs across participants, so the spatial distribution in (a) reflects pooled data, whereas the response diversity is visualized in the within-contact comparisons in (b). *p < 0.05, ***p < 0.001.

After mapping the neural responses to syllabic, phrasal, and sentential structures in speech, we then examined the shared and distinct neural clusters responsive to linguistic structures at different levels. We highlighted the contacts that showed low-frequency neural entrainment to higher-order structures at phrase-level (*f*_phr_+) or sentence-level (*f*_sen_+) rates but did not respond at the syllable-level (*f*_syl_-) Fig. 4a). Most of neural clusters were localized in the fronto-temporal region and the temporo-parietal junction (TPJ), with some also dispersed in a distributed manner. The neural clusters that responded solely to higher-order linguistic structures (as shown in Fig. 4b, Selective) indicate that there may be certain brain mechanisms specialized for internal construction of abstract linguistic structures independent of syllabic rate fluctuations. Additionally, we pinpointed a separate population of neural clusters that were sensitive to both higher-order linguistic structures and basic syllabic processing (as shown in Fig. 4b, Non-selective, *f*_syl_+), demonstrating that particular neural clusters can handle multiple levels of linguistic information in continuous speech simultaneously.

Mixed-effects logistic regression analysis on response selectivity (*f*+/*f-*) revealed a significant region × hemisphere × linguistic level interaction (likelihood ratio test: χ²(8) = 17.72, *p* = 0.023), indicating that the probability of observing structure-selective responses depends jointly on cortical region, hemisphere, and linguistic timescale. Follow-up hemisphere-specific analyses showed significant region × linguistic level interactions in both hemispheres (left: χ²(8) = 28.15, *p* < 0.001; right: χ²(8) = 44.60, *p* < 0.001), consistent with the graded and region-specific tracking patterns observed in the linear mixed-effects analyses. Post-hoc comparisons confirmed that contacts in the auditory cortex, particularly HG and STG, were the most likely to exhibit syllable-level sensitivity, whereas higher-order temporal and frontal regions showed increased likelihood of phrase- and sentence-level sensitivity. Together, these results revealed a spatially organized pattern of neural sensitivity to syllabic, phrasal, and sentential structures.

### 3.2 High-gamma activity entrained to speech structures

**Fig. 5.**
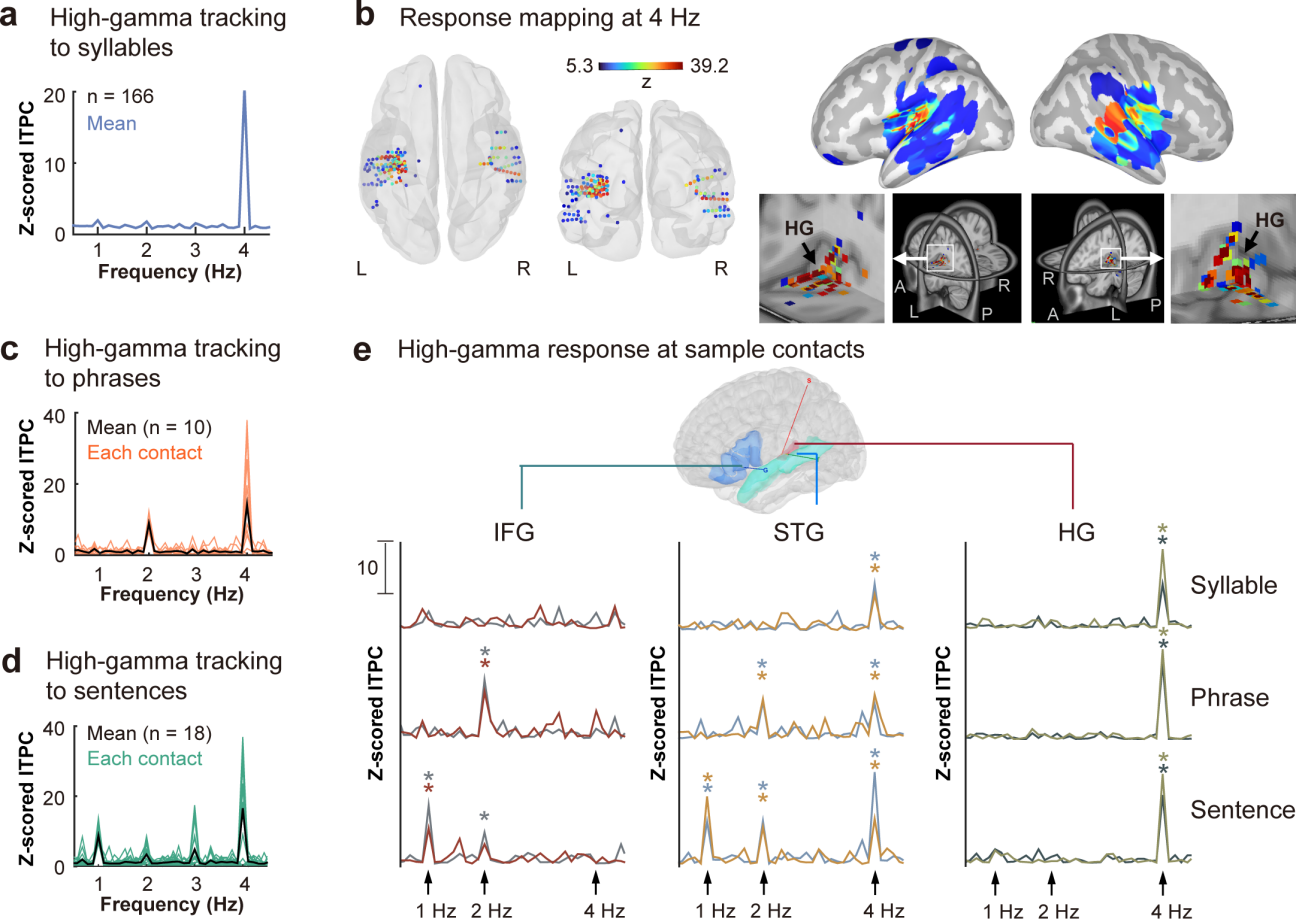
High-gamma neural tracking to speech structures. (a) Significant syllabic rate tracking at 4 Hz was observed in 166 contacts after thresholding (corrected *p* < 0.001). (b) Response mapping at 4 Hz showed distribution primarily centering in bilateral HG. (c) High gamma tracking responses to phrases in each responsive contact (in yellow) and their averaged responses (in black). (d) High gamma tracking responses to sentences in each responsive contact (in blue) and their averaged responses (in black). (e) high-gamma response at sample contacts localized in the left fronto-temporal regions of IFG (MNI coordinates of the contacts: [-49, 25, -5], [-53, 25, -4]), STG (MNI coordinates of the contacts: [-55, -16, 9], [-59, -17, 9]), and HG (MNI coordinates of the contacts: [-39, -17, 6], [-39, -18, 10]). Contacts within the same brain areas were adjacent to each other, coming from the same electrode and subject. In HG, consistent robust high-gamma tracking at 4 Hz was observed irrespective of whether syllables, phrases, or sentences were being processed. STG contacts exhibited tracking responses to both syllabic and higher linguistic unit rates, while IFG contacts only responded to the construction of higher-order linguistic structures, *p < 0.05. Time-series plots of these representative contacts showed periodical changing patterns (Supplementary Fig. S4).

Contacts displayed significant high-gamma activities entrained to speech structures were identified after applying a threshold criteria of z = 5.29 (corresponding to a *p* value of 0.001 in a two-tailed normalized distribution, adjusted by Bonferroni correction) to z-scored ITPC. A total of 166 contacts showed significant tracking responses at the syllabic rate of 4 Hz (Fig. 5a), with localization predominantly centered in the primary auditory cortex (HG) as depicted in Figure 5b. At the phrase-level of 2 Hz, 10 contacts displayed significant tracking responses (Fig. 5c), while 18 contacts showed significant tracking responses to sentential structures (Fig. 5e). Contacts responsive to phrasal and sentential rates were primarily located in the fronto-temporal region, including primary auditory cortex, secondary auditory cortex, inferior frontal lobe. Detailed AAL labels and MNI coordinates of these responsive contacts were summarized in Supplementary Materials (Supplementary Table S5).

In Figure 5d, we demonstrated the high-gamma spectral responses to syllables, phrases, and sentences at sample contacts. The spectral tracking of six contacts within the left fronto-temporal network was shown, including two adjacent contacts in the IFG, STG, and HG, respectively. The contacts in HG showed notable tracking responses at 4 Hz, irrespective of whether syllables, phrases, or sentences were being processed. In STG, both syllabic rate tracking and phrasal/sentential tracking were observed. Intriguingly, the IFG contacts exclusively responded to higher-order linguistic structures, showing no tracking responses to the syllabic rate any more. The hierarchical nature of this neural network, extending the primary auditory cortex to the secondary auditory cortex and then to the frontal lobe, indicates the spatially distinct high-gamma tracking mechanism underlying the formation of abstract linguistic representations and processing amplitude fluctuations during speech comprehension.

Among all the contacts recorded, a notable difference is observed in response percentages between low-frequency (n = 502 at 4 Hz, n = 143 at 2 Hz, and n = 125 at 1 Hz) and high-gamma neural tracking (n = 166 at 4 Hz, n = 10 at 2 Hz, and n = 18 at 1 Hz). This difference is also evident in the neural decoding performance using low-frequency and high-gamma signals in the left fronto-temporal network, where low-frequency signals exhibit higher decoding efficiency compared to high-frequency signals (refer to Supplementary Materials, Supplementary Fig. S3).

## 4 Discussion

This study made use of clinically implanted intracranial sEEG recordings to examine the cortical regions involved in tracking hierarchical linguistic structures. The results revealed robust fronto-temporal neural tracking at 1 Hz for syllabic rhythm, at 2 Hz for phrasal rhythm, and at 4 Hz for sentential rhythm. During both low-frequency and high-gamma tracking, the primary auditory cortex demonstrated the strongest involvement at the syllable level, and the superior temporal region and inferior frontal region were found to be engaged in higher-level processing of phrasal and sentential structures. Using depth electrodes, we observed that many contacts with syllabic-rate tracking were located in Heschl’s gyrus, whereas contacts in the outer superior temporal gyrus showed less prominent responses. Moreover, compared to low-frequency tracking, high-gamma responses were found to be more sparsely distributed, predominantly situated in a select number of fronto-temporal sites. The spatial differentiation of neural tracking across linguistic structures reflects differences in cortical sensitivity to distinct linguistic timescales, indicating that separate brain regions engage preferentially with different rhythmic components of speech.

Our study replicates and extends the work of Ding et al. (2016), with modifications to the experimental design. While Ding et al. (2016) focused on testing concurrent tracking responses across three linguistic levels using sentence stimuli and acoustic control, our study expanded the speech stimuli to include syllables, phrases, and sentences. This adjustment allowed us to eliminate harmonic responses at 2 Hz and 4 Hz that could potentially interfere with the assessment of phrasal and syllabic responses. Furthermore, by incorporating three types of speech stimuli, we were able to observe distinct response patterns across electrodes, transitioning from syllable sequences to sentence sequences. This comprehensive approach provided a more thorough examination of the neural mapping involved in hierarchical linguistic construction.

Our study identified distinct populations of electrodes that respond to syllabic, phrasal, and sentential structures in spatially differentiated patterns, as illustrated in Figure 3. These findings align with a previous study reported by Ding et al. (2016), and also consistent with the MEG source estimation outcomes reported by Sheng et al. (2019). Furthermore, our study recorded neural activities directly from the primary auditory cortex using depth electrode recordings, documenting the activation pattern changing from the primary auditory cortex to the secondary auditory cortex. Collectively, these results are consistent with the work by Keshishian et al. (2023), which proposes that linguistic feature encoding follows a joint and hierarchical organization in the human auditory cortex.

Ding et al. (2016) previously reported significant high-gamma responses to sentential structures in the posterior and middle STG and left IFG, as well as to phrasal structures in bilateral posterior STG. Similarly, our investigation identified electrodes exhibiting significant high-gamma tracking responses in the STG and IFG at the sentence level, with additional responsive electrodes detected in the left HG, middle, and inferior temporal regions. Furthermore, significant responses were observed in temporal and frontal regions at the phrasal level. Overall, our study identified more extensive and distributed brain regions exhibiting significantly higher high-gamma tracking responses to higher-level linguistic structures compared to the Ding et al. (2016). Within these responsive regions, representative electrodes in the HG, STG, and IFG demonstrated distinct response patterns to the three linguistic levels, as illustrated in Figure 5. The IFG was implicated in the construction of sentences and phrases, while HG responses were associated with the basic syllabic rhythms. Furthermore, electrodes in the STG displayed simultaneous responses across all linguistic hierarchies. These findings indicate a possible internal transformation mechanism in the left fronto-temporal region that is specialized in concurrently representing abstract linguistic structures. Compared to low-frequency tracking, the high-gamma activities emphasize more localized computations and reveal non-overlapping patterns of activation, reflecting distinct neural dynamics such as spiking-related activity and local encoding processes (Liégeois-Chauvel et al., 2022; Luo et al., 2024).

Previous intracranial EEG studies have provided valuable insights into the neural mechanisms of speech and language. Early sEEG and ECoG work has demonstrated the specialization of the left auditory cortex for speech perception and syllabic processing (Liégeois-Chauvel et al., 1999; 2004), as well as asymmetries in temporal resolution between hemispheres (Morillon et al., 2012). Others have explored the temporal envelope encoding in the auditory cortex (e.g., Liégeois-Chauvel et al., 2004), hippocampal and auditory contributions to statistical speech segmentation (Ramos-Escobar et al., 2022), and efforts to decode imagined and perceived speech using intracranial signals (Martin et al., 2019). Critically, Nelson et al. (2017) used intracranial recordings and visual word-by-word sentence presentation to reveal that high-gamma activity increased with incremental word input and showed sudden decreases when syntactic phrases could be merged, providing key evidence for a neurophysiological basis of syntactic structure building consistent with a bottom-up parsing mechanism. These findings strongly support the existence of cortical computations implementing hierarchical linguistic operations. Our study complements this body of work by employing a frequency-tagging paradigm to reveal how syllables, phrases, and sentences are tracked at distinct frequencies across fronto-temporal cortical regions, contributing further evidence for hierarchical linguistic organization in the human brain.

The current study has some limitations that need to be addressed. During the experimental task, participants were instructed to focus on the speech stream without any behavioral indicators. The decision to avoid complex experimental tasks was influenced by the clinical environment and time constraints, leading to a lack of objective evaluation of subject’s auditory attention state. It has been suggested that attentional and consciousness states significantly influence the frequency-tagged neural tracking responses to target speech, particularly with regards to the construction of higher-order linguistic structures, including phrases and sentences (Ding et al., 2018; Makov et al., 2017). The effect of selective attention on brain’s representation of speech appears to manifest in intracranial neural dynamics, as evidenced by fluctuations in low-frequency and high-frequency responses (Golumbic et al., 2013; Raghavan et al., 2023). Hence, it is essential for forthcoming investigations to monitor participants’ attentional states using behavioral testing, such as lexical or semantic judgements (Ding et al., 2016; Sheng et al., 2019), during intracranial recordings to tackle this issue. Moreover, the current study focuses on frequency-tagged tracking responses in the low-frequency and high-gamma envelope spectrum. In future work, it would be of interest to examine cross-frequency interactions through phase-amplitude coupling across different frequency bands, which may help reveal underlying mechanisms such as feature binding and speech representation. In addition, intracranial electrodes are implanted for clinical purposes, leading to uneven spatial coverage across participants. To provide transparency regarding the composition of the sEEG dataset, we report the number of unique participants contributing to each set of responsive contacts in the Supplementary Materials (Supplementary Tables S5 and S6). These summaries show that responsive contacts are unevenly distributed across atlas-defined regions, but in most cases are contributed by multiple participants rather than driven by a single individual. Importantly, by applying linear mixed-effects models that account for subject- and contact-level variability, we were able to formally assess response patterns despite uneven spatial coverage, confirming the robustness of tracking differences across cortical regions and linguistic levels.

Unlike Ding et al. (2016), who used power analysis, we employed ITPC to capture neural tracking of linguistic rhythms by focusing on phase consistency across trials rather than spectral power. This method is particularly suited for frequency-tagging paradigms, emphasizing temporal alignment of neural responses to rhythmic stimuli. Compared to power-based measures, ITPC is less affected by amplitude variability and noise, offering a more robust index of neural entrainment, consistent with prior studies (Ding et al., 2018; Gui et al., 2020; Lu et al., 2023). In addition, because our stimuli lacked natural prosodic cues, speech decoding likely relied more heavily on internal mechanisms to segment and interpret the linguistic structures, similar to the demands observed in artificial language learning paradigms (Saffran et al., 2001). Future studies could compare prosodically natural versus rhythmically controlled speech to assess how neural tracking differs when listeners rely on acoustic cues versus internal linguistic knowledge for structure segmentation. Relatedly, our results showed that low-level acoustic tracking at the syllabic rate was bilaterally distributed across the primary auditory cortex, and tracking of phrasal and sentential structures also involved right-hemisphere regions. This may reflect the nature of the frequency-tagging paradigm, which introduces rhythmic changes at multiple time scales and may lead to perceived prosodic structuring. These temporal regularities could engage right-lateralized mechanisms known to support prosodic processing, as suggested by previous studies (Gandour et al., 2004; Homae et al., 2006). Nevertheless, caution should be taken when interpreting lateralization patterns, as the number and distribution of electrodes across hemispheres were inherently uneven in intracranial recordings.

In summary, our study provides new sEEG evidence for the intracranial mapping of hierarchical linguistic structure tracking in speech, using a frequency-tagging approach with high spatial resolution. By employing a frequency-tagging paradigm to separate linguistic structures from lower- to higher-levels, we revealed hierarchically organized and spatially differentiated brain regions responsible for the distinct levels of the hierarchical linguistic structures.

## Supplementary Material

Supplementary Material

## Data Availability

Data supporting the study findings and codes for data analyses are available from the corresponding author upon written request. The analysis code is also publicly available at: https://osf.io/ad6qc

## References

[IMAG.a.1158-b1] Aron, O., Jonas, J., Colnat-Coulbois, S., & Maillard, L. (2021). Language mapping using stereo electroencephalography: A review and expert opinion. Frontiers in Human Neuroscience, 15, 619521. 10.3389/fnhum.2021.619521 33776668 PMC7987679

[IMAG.a.1158-b2] Berwick, R. C., Friederici, A. D., Chomsky, N., & Bolhuis, J. J. (2013). Evolution, brain, and the nature of language. Trends in Cognitive Sciences, 17(2), 89–98. 10.1016/j.tics.2012.12.002 23313359

[IMAG.a.1158-b3] Chomsky, N. (1957). Syntactic structures. De Gruyter Mouton. 10.1515/9783112316009

[IMAG.a.1158-b4] Cohen, M. X. (2014). Analyzing neural time series data: Theory and practice. Cambridge: MIT Press. 10.7551/mitpress/9609.001.0001

[IMAG.a.1158-b5] Ding, N., Melloni, L., Zhang, H., Tian, X., & Poeppel, D. (2016). Cortical tracking of hierarchical linguistic structures in connected speech. Nature Neuroscience, 19(1), 158–164. 10.1038/nn.4186 26642090 PMC4809195

[IMAG.a.1158-b6] Ding, N., Pan, X., Luo, C., Su, N., Zhang, W., & Zhang, J. (2018). Attention is required for knowledge-based sequential grouping: Insights from the integration of syllables into words. Journal of Neuroscience, 38(5), 1178–1188. 10.1523/jneurosci.2606-17.2017 29255005 PMC6596269

[IMAG.a.1158-b7] Ding, N., & Simon, J. Z. (2013). Adaptive temporal encoding leads to a background-insensitive cortical representation of speech. Journal of Neuroscience, 33(13), 5728–5735. 10.1523/jneurosci.5297-12.2013 23536086 PMC3643795

[IMAG.a.1158-b8] Doelling, K. B., Arnal, L. H., Ghitza, O., & Poeppel, D. (2014). Acoustic landmarks drive delta–theta oscillations to enable speech comprehension by facilitating perceptual parsing. Neuroimage, 85, 761–768. 10.1016/j.neuroimage.2013.06.035 23791839 PMC3839250

[IMAG.a.1158-b9] Gandour, J., Tong, Y., Wong, D., Talavage, T., Dzemidzic, M., Xu, Y., Li, X., & Lowe, M. (2004). Hemispheric roles in the perception of speech prosody. NeuroImage, 23(1), 344–357. 10.1016/j.neuroimage.2004.06.004 15325382

[IMAG.a.1158-b10] Golumbic, E. M. Z., Ding, N., Bickel, S., Lakatos, P., Schevon, C. A., McKhann, G. M., Goodman, R. R., Emerson, R., Mehta, A. D., & Simon, J. Z. (2013). Mechanisms underlying selective neuronal tracking of attended speech at a “cocktail party.” Neuron, 77(5), 980–991. 10.1016/j.neuron.2012.12.037 23473326 PMC3891478

[IMAG.a.1158-b11] Gui, P., Jiang, Y., Zang, D., Qi, Z., Tan, J., Tanigawa, H., Jiang, J., Wen, Y., Xu, L., & Zhao, J. (2020). Assessing the depth of language processing in patients with disorders of consciousness. Nature Neuroscience, 23(6), 761–770. 10.1038/s41593-020-0639-1 32451482

[IMAG.a.1158-b12] Har-shai Yahav, P., & Zion Golumbic, E. (2021). Linguistic processing of task-irrelevant speech at a cocktail party. Elife, 10, e65096. 10.7554/elife.65096 33942722 PMC8163500

[IMAG.a.1158-b13] Homae, F., Watanabe, H., Nakano, T., Asakawa, K., & Taga, G. (2006). The right hemisphere of sleeping infant perceives sentential prosody. Neuroscience Research, 54(4), 276–280. 10.1016/j.neures.2005.12.006 16427714

[IMAG.a.1158-b14] Keshishian, M., Akkol, S., Herrero, J., Bickel, S., Mehta, A. D., & Mesgarani, N. (2023). Joint, distributed and hierarchically organized encoding of linguistic features in the human auditory cortex. Nature Human Behaviour, 7(5), 740–753. 10.1038/s41562-023-01520-0 PMC10417567 36864134

[IMAG.a.1158-b15] Kojima, K., Brown, E. C., Rothermel, R., Carlson, A., Fuerst, D., Matsuzaki, N., Shah, A., Atkinson, M., Basha, M., & Mittal, S. (2013). Clinical significance and developmental changes of auditory-language-related gamma activity. Clinical Neurophysiology, 124(5), 857–869. 10.1016/j.clinph.2012.09.031 23141882 PMC3577943

[IMAG.a.1158-b16] Liégeois-Chauvel, C., De Graaf, J. B., Laguitton, V., & Chauvel, P. (1999). Specialization of left auditory cortex for speech perception in man depends on temporal coding. Cerebral Cortex, 9(5), 484–496. 10.1093/cercor/9.5.484 10450893

[IMAG.a.1158-b17] Liégeois-Chauvel, C., Dubarry, A.-S., Wang, I., Chauvel, P., Gonzalez-Martinez, J. A., & Alario, F.-X. (2022). Inter-individual variability in dorsal stream dynamics during word production. European Journal of Neuroscience, 56(7), 5070–5089. 10.1111/ejn.15807 35997580 PMC9804493

[IMAG.a.1158-b18] Liégeois-Chauvel, C., Lorenzi, C., Trébuchon, A., Régis, J., & Chauvel, P. (2004). Temporal envelope processing in the human left and right auditory cortices. Cerebral Cortex, 14(7), 731–740. 10.1093/cercor/bhh033 15054052

[IMAG.a.1158-b19] Lizarazu, M., Carreiras, M., Bourguignon, M., Zarraga, A., & Molinaro, N. (2021). Language proficiency entails tuning cortical activity to second language speech. Cerebral Cortex, 31(8), 3820–3831. 10.1093/cercor/bhab051 33791775

[IMAG.a.1158-b20] Lu, L., Deng, Y., Xiao, Z., Jiang, R., & Gao, J.-H. (2023). Neural signatures of hierarchical linguistic structures in second language listening comprehension. Eneuro, 10(6), ENEURO.0346-22.2023. https://www.eneuro.org/content/10/6/ENEURO.0346-22.2023.abstract 10.1523/ENEURO.0346-22.2023 PMC10294774 37328296

[IMAG.a.1158-b21] Lu, L., Wang, Q., Sheng, J., Liu, Z., Qin, L., Li, L., & Gao, J.-H. (2019). Neural tracking of speech mental imagery during rhythmic inner counting. elife, 8, e48971. 10.7554/elife.48971 PMC6805153 31635693

[IMAG.a.1158-b22] Luo, H., & Poeppel, D. (2007). Phase patterns of neuronal responses reliably discriminate speech in human auditory cortex. Neuron, 54(6), 1001–1010. 10.1016/j.neuron.2007.06.004 17582338 PMC2703451

[IMAG.a.1158-b23] Luo, L., Wang, X., Lu, J., Chen, G., Luan, G., Li, W., Wang, Q., & Fang, F. (2024). Local field potentials, spiking activity, and receptive fields in human visual cortex. Science China Life Sciences, 67(3), 543–554. 10.1007/s11427-023-2436-x 37957484

[IMAG.a.1158-b24] Makov, S., Sharon, O., Ding, N., Ben-Shachar, M., Nir, Y., & Golumbic, E. Z. (2017). Sleep disrupts high-level speech parsing despite significant basic auditory processing. Journal of Neuroscience, 37(32), 7772–7781. 10.1523/jneurosci.0168-17.2017 28626013 PMC6596654

[IMAG.a.1158-b25] Martin, S., Millán, J. D. R., Knight, R. T., & Pasley, B. N. (2019). The use of intracranial recordings to decode human language: Challenges and opportunities. Brain and Language, 193, 73–83. 10.1016/j.bandl.2016.06.003 27377299 PMC5203979

[IMAG.a.1158-b26] Molinaro, N., & Lizarazu, M. (2018). Delta(but not theta)‐band cortical entrainment involves speech‐specific processing. European Journal of Neuroscience, 48(7), 2642–2650. 10.1111/ejn.13811 29283465

[IMAG.a.1158-b27] Morillon, B., Liégeois-Chauvel, C., Arnal, L. H., Bénar, C.-G., & Giraud, A.-L. (2012). Asymmetric function of theta and gamma activity in syllable processing: An intra-cortical study. Frontiers in Psychology, 3, 248. 10.3389/fpsyg.2012.00248 22833730 PMC3400438

[IMAG.a.1158-b28] Nelson, M. J., El Karoui, I., Giber, K., Yang, X., Cohen, L., Koopman, H., Cash, S. S., Naccache, L., Hale, J. T., Pallier, C., & Dehaene, S. (2017). Neurophysiological dynamics of phrase-structure building during sentence processing. Proceedings of the National Academy of Sciences, 114(18), E3669–E3678. 10.1073/pnas.1701590114 PMC5422821 28416691

[IMAG.a.1158-b29] Raghavan, V. S., O’Sullivan, J., Bickel, S., Mehta, A. D., & Mesgarani, N. (2023). Distinct neural encoding of glimpsed and masked speech in multitalker situations. PLoS Biology, 21(6), e3002128. 10.1371/journal.pbio.3002128 37279203 PMC10243639

[IMAG.a.1158-b30] Ramos-Escobar, N., Mercier, M., Trébuchon-Fonséca, A., Rodriguez-Fornells, A., François, C., & Schön, D. (2022). Hippocampal and auditory contributions to speech segmentation. Cortex, 150, 1–11. 10.1016/j.cortex.2022.01.017 35305505

[IMAG.a.1158-b31] Ray, S., & Maunsell, J. H. (2011). Different origins of gamma rhythm and high-gamma activity in macaque visual cortex. PLoS Biology, 9(4), e1000610. 10.1371/journal.pbio.1000610 21532743 PMC3075230

[IMAG.a.1158-b32] Reetzke, R., Gnanateja, G. N., & Chandrasekaran, B. (2021). Neural tracking of the speech envelope is differentially modulated by attention and language experience. Brain and Language, 213, 104891. 10.1016/j.bandl.2020.104891 33290877 PMC7856208

[IMAG.a.1158-b33] Saffran, J. R., Senghas, A., & Trueswell, J. C. (2001). The acquisition of language by children. Proceedings of the National Academy of Sciences, 98(23), 12874–12875. 10.1073/pnas.231498898 PMC60790 11687645

[IMAG.a.1158-b34] Sheng, J., Zheng, L., Lyu, B., Cen, Z., Qin, L., Tan, L. H., Huang, M.-X., Ding, N., & Gao, J.-H. (2019). The cortical maps of hierarchical linguistic structures during speech perception. Cerebral Cortex, 29(8), 3232–3240. 10.1093/cercor/bhy191 30137249

[IMAG.a.1158-b35] Woolnough, O., Donos, C., Murphy, E., Rollo, P. S., Roccaforte, Z. J., Dehaene, S., & Tandon, N. (2023). Spatiotemporally distributed frontotemporal networks for sentence reading. Proceedings of the National Academy of Sciences United States of America, 120(17), e2300252120. https://www.pnas.org/doi/abs/10.1073/pnas.2300252120 10.1073/pnas.2300252120 PMC10151604 37068244

