# Supplementary material for: Intracranial mapping of linguistic structure building during listening speech comprehension
Source: Imaging Neurosci (Camb). 2026 Mar 4;4:IMAG.a.1158. doi: 10.1162/IMAG.a.1158 (PMC12961306; doi:10.1162/IMAG.a.1158)
Supplement: Supplementary Material [file IMAG.a.1158_supp.pdf]

Supplementary materials

Speech Materials

100 four-syllable (two-phrase) Chinese sentences used in the current study are listed below.

Sentence pool A

朋友请客	汽车拐弯	青草发芽	飞机降落	游客爬山
母鸡下蛋	灯泡发光	英雄救火	骏马奔驰	农民种菜
太阳落山	鲸鱼喷水	狮子打架	公司开张	苹果成熟
绵羊吃草	护士打针	法官判案	开水沸腾	观众鼓掌
老牛耕地	行人过街	鸭子游泳	士兵战斗	厨师做饭
老师讲课	树木生长	外公看报	渔民划船	和尚念经
小孩哭泣	熊猫睡觉	蝴蝶飞舞	导游讲解	学生写字
医生看病	军队撤退	演员跳舞	兄弟吵架	小偷逃走
猎豹奔跑	蜘蛛结网	叛徒告密	冰雪融化	婴孩啼哭
轮船启航	乌龟爬行	剪刀生锈	祖父下棋	电脑开机

Sentence pool B

阿姨做饭	病人吃药	财主讨债	车辆行驶	尘土飞扬
敌军撤退	鳄鱼冬眠	歌手演唱	公鸡打鸣	顾客结账
管道破裂	河水结冰	会议闭幕	火车晚点	火山爆发
机器运行	计划成功	警察抓人	军队训练	蜡烛燃烧
老人散步	老师提问	猎豹捕食	领导退休	律师辩护
梅花绽放	猛虎怒吼	蜜蜂飞舞	皮球泄气	强盗打劫
樵夫砍柴	青蛙游泳	山洪暴发	时间流逝	实验成功
书籍出版	树枝抖动	司机开车	蜗牛爬行	乌云飘走
小孩玩耍	兄弟聊天	雄鹰翱翔	学生考试	学校关闭
雪花飘舞	叶子变黄	游客观赏	陨石坠落	专家讨论

Statistical results of all pairwise post-hoc comparisons in LMMs

In each hemisphere, detailed post-hoc comparisons between linguistic levels and brain regions are summarized below, with corresponding plots provided for visualization., Syl., syllable; Phr, phrase; Sen, sentence; HG, Helsch Gyrus; IFG, inferior frontal gyrus; STG, superior temporal gyrus; MTG, middle temporal gyrus; ITG, inferior temporal gyrus. $^{*}p < 0.05$, $^{**}p < 0.01$, $^{***}p < 0.001$, with FDR correction.

Left Hemisphere

Table S1 Comparisons between linguistic levels in the left hemisphere

Contrast	Region	Estimate	SE	df	lowerCL	upperCL	t	p value
Syl - Phr	IFG	3.12	1.22	782.1	0.21	6.04	2.57	0.031*
Syl - Sen	IFG	1.97	1.22	782.1	-0.95	4.88	1.62	0.159
Phr - Sen	IFG	-1.16	1.22	782.1	-4.07	1.76	-0.95	0.341
Syl - Phr	HG	24.67	1.60	782.1	20.82	28.51	15.39	<0.001***
Syl - Sen	HG	26.75	1.60	782.1	22.91	30.60	16.69	<0.001***
Phr - Sen	HG	2.08	1.60	782.1	-1.76	5.93	1.30	0.194
Syl - Phr	ITG	1.73	0.93	782.1	-0.51	3.96	1.85	0.096
Syl - Sen	ITG	1.96	0.93	782.1	-0.27	4.20	2.11	0.096
Phr - Sen	ITG	0.24	0.93	782.1	-2.00	2.47	0.25	0.801
Syl - Phr	MTG	4.34	0.58	782.1	2.96	5.73	7.54	<0.001***
Syl - Sen	MTG	4.78	0.58	782.1	3.40	6.17	8.31	<0.001***
Phr - Sen	MTG	0.44	0.58	782.1	-0.94	1.82	0.77	0.444
Syl - Phr	STG	16.20	0.88	782.1	14.10	18.30	18.50	<0.001***
Syl - Sen	STG	18.45	0.88	782.1	16.35	20.55	21.06	<0.001***
Phr - Sen	STG	2.24	0.88	782.1	0.14	4.35	2.56	0.011*

Table S2 Comparisons between brain regions in the left hemisphere

Contrast	Level	Estimate	SE	df	lowerCL	upperCL	t	p value
IFG - HG	Syl	-25.20	1.59	988.9	-29.66	-20.74	-15.89	<0.001***
IFG - ITG	Syl	0.76	1.28	558.2	-2.84	4.35	0.59	0.554
IFG - MTG	Syl	-2.59	1.15	429.4	-5.83	0.65	-2.26	0.027*
IFG - STG	Syl	-19.05	1.21	768.6	-22.47	-15.63	-15.68	<0.001***
HG - ITG	Syl	25.95	1.42	1125.5	21.96	29.95	18.26	<0.001***
HG - MTG	Syl	22.61	1.31	1120.8	18.92	26.30	17.23	<0.001***
HG - STG	Syl	6.15	1.39	1119.3	2.24	10.07	4.42	<0.001***
ITG - MTG	Syl	-3.35	0.84	1119.9	-5.72	-0.97	-3.97	<0.001***
ITG - STG	Syl	-19.80	1.01	1073.5	-22.63	-16.97	-19.69	<0.001***
MTG - STG	Syl	-16.45	0.83	1012.0	-18.80	-14.11	-19.71	<0.001***
IFG - HG	Phr	-3.65	1.59	988.9	-8.11	0.81	-2.30	0.054
IFG - ITG	Phr	-0.64	1.28	558.2	-4.24	2.96	-0.50	0.617
IFG - MTG	Phr	-1.37	1.15	429.4	-4.61	1.87	-1.20	0.291
IFG - STG	Phr	-5.97	1.21	768.6	-9.39	-2.55	-4.91	<0.001***
HG - ITG	Phr	3.01	1.42	1125.5	-0.98	7.01	2.12	0.068

HG - MTG	Phr	2.28	1.31	1120.8	-1.41	5.97	1.74	0.137
HG - STG	Phr	-2.31	1.39	1119.3	-6.23	1.60	-1.66	0.138
ITG - MTG	Phr	-0.73	0.84	1119.9	-3.10	1.64	-0.87	0.428
ITG - STG	Phr	-5.33	1.01	1073.5	-8.16	-2.50	-5.30	<0.001***
MTG - STG	Phr	-4.60	0.83	1012.0	-6.94	-2.25	-5.50	<0.001***
IFG - HG	Sen	-0.41	1.59	988.9	-4.87	4.05	-0.26	0.842
IFG - ITG	Sen	0.75	1.28	558.2	-2.84	4.35	0.59	0.783
IFG - MTG	Sen	0.23	1.15	429.4	-3.01	3.46	0.20	0.842
IFG - STG	Sen	-2.56	1.21	768.6	-5.98	0.86	-2.11	0.117
HG - ITG	Sen	1.16	1.42	1125.5	-2.83	5.16	0.82	0.783
HG - MTG	Sen	0.64	1.31	1120.8	-3.05	4.33	0.49	0.783
HG - STG	Sen	-2.15	1.39	1119.3	-6.07	1.76	-1.55	0.305
ITG - MTG	Sen	-0.53	0.84	1119.9	-2.90	1.85	-0.62	0.783
ITG - STG	Sen	-3.32	1.01	1073.5	-6.15	-0.49	-3.30	0.005**
MTG - STG	Sen	-2.79	0.83	1012.0	-5.14	-0.44	-3.34	0.005**

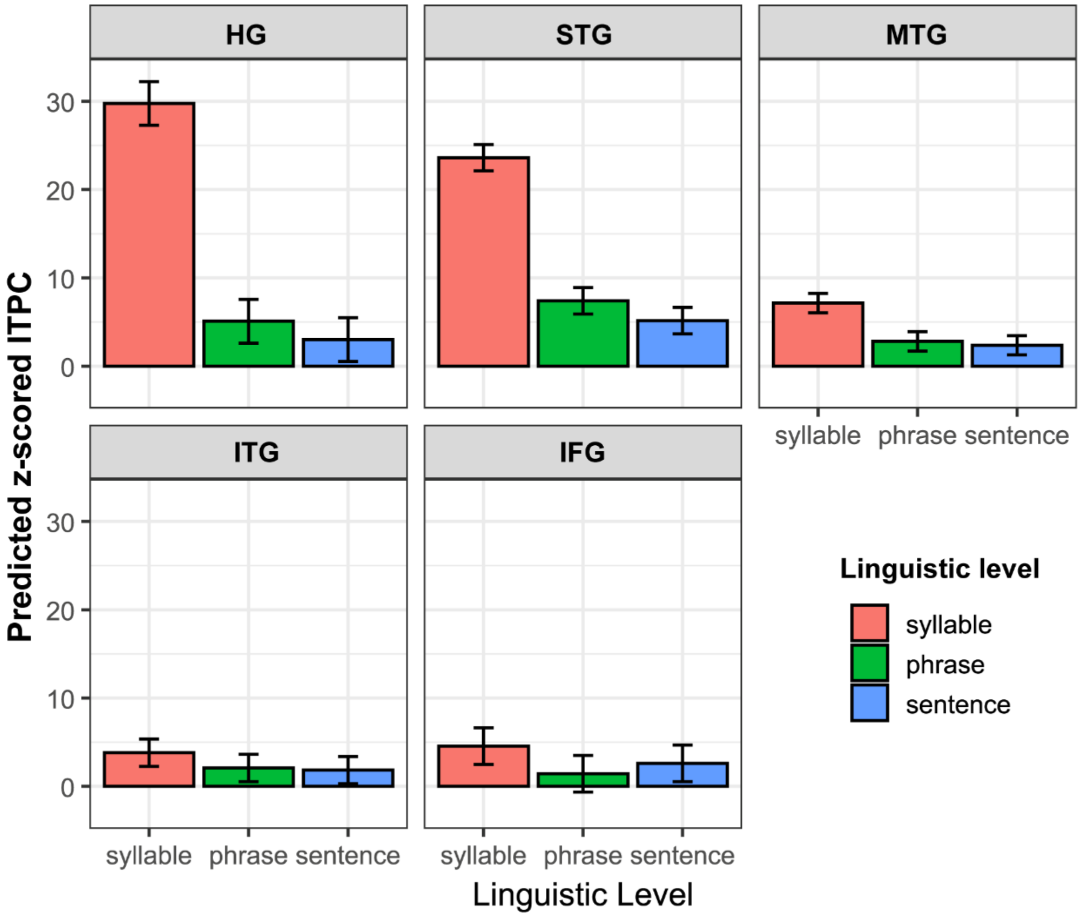

Figure S1 Estimated marginal means of z-scored ITPC across linguistic levels and regions from linear mixed-effects models (region × linguistic level) in the left hemisphere. Error bars indicated 95% confidence intervals (CIs).

Right Hemisphere

Table S3 Comparisons between linguistic levels in the right hemisphere

Contrast	Region	Estimate	SE	df	lowerCL	upperCL	t	p value
Syl - Phr	IFG	2.10	1.32	298.4	-1.07	5.27	1.59	0.169
Syl - Sen	IFG	-1.60	1.32	298.4	-4.77	1.57	-1.21	0.226
Phr - Sen	IFG	-3.70	1.32	298.4	-6.87	-0.52	-2.81	0.016*
Syl - Phr	HG	21.53	3.16	298.4	13.92	29.14	6.81	<0.001***
Syl - Sen	HG	26.55	3.16	298.4	18.94	34.16	8.40	<0.001***
Phr - Sen	HG	5.03	3.16	298.4	-2.58	12.64	1.59	0.113
Syl - Phr	ITG	5.61	0.87	298.4	3.52	7.70	6.46	<0.001***
Syl - Sen	ITG	5.48	0.87	298.4	3.39	7.57	6.31	<0.001***
Phr - Sen	ITG	-0.13	0.87	298.4	-2.22	1.96	-0.15	0.884
Syl - Phr	MTG	6.31	0.86	298.4	4.24	8.39	7.34	<0.001***
Syl - Sen	MTG	5.32	0.86	298.4	3.25	7.39	6.19	<0.001***
Phr - Sen	MTG	-0.99	0.86	298.4	-3.06	1.08	-1.15	0.250
Syl - Phr	STG	11.42	1.91	298.4	6.83	16.01	5.99	<0.001***
Syl - Sen	STG	20.63	1.91	298.4	16.04	25.22	10.82	<0.001***
Phr - Sen	STG	9.21	1.91	298.4	4.62	13.80	4.83	<0.001***

Table S4 Comparisons between brain regions in the right hemisphere

Contrast	Level	Estimate	SE	df	lowerCL	upperCL	t	p value
IFG - HG	Syl	-23.44	2.51	444.4	-30.51	-16.37	-9.35	<0.001***
IFG - ITG	Syl	-2.97	1.55	297.8	-7.35	1.41	-1.92	0.070
IFG - MTG	Syl	-3.53	1.62	266.0	-8.12	1.06	-2.18	0.043*
IFG - STG	Syl	-22.88	2.01	372.8	-28.55	-17.21	-11.39	<0.001***
HG - ITG	Syl	20.47	2.38	441.2	13.74	27.19	8.59	<0.001***
HG - MTG	Syl	19.91	2.41	442.9	13.11	26.70	8.27	<0.001***
HG - STG	Syl	0.56	2.68	441.3	-7.01	8.13	0.21	0.835
ITG - MTG	Syl	-0.56	0.92	434.9	-3.16	2.04	-0.61	0.601
ITG - STG	Syl	-19.91	1.49	438.9	-24.12	-15.70	-13.34	<0.001***
MTG - STG	Syl	-19.35	1.53	437.3	-23.66	-15.03	-12.65	<0.001***
IFG - HG	Phr	-4.01	2.51	444.4	-11.08	3.06	-1.60	0.158
IFG - ITG	Phr	0.54	1.55	297.8	-3.84	4.92	0.35	0.808
IFG - MTG	Phr	0.68	1.62	266.0	-3.91	5.27	0.42	0.808
IFG - STG	Phr	-13.56	2.01	372.8	-19.23	-7.88	-6.75	<0.001***
HG - ITG	Phr	4.55	2.38	441.2	-2.17	11.28	1.91	0.095
HG - MTG	Phr	4.69	2.41	442.9	-2.10	11.49	1.95	0.095
HG - STG	Phr	-9.55	2.68	441.3	-17.12	-1.98	-3.56	0.001**
ITG - MTG	Phr	0.14	0.92	434.9	-2.46	2.74	0.15	0.878
ITG - STG	Phr	-14.10	1.49	438.9	-18.31	-9.89	-9.45	<0.001***
MTG - STG	Phr	-14.24	1.53	437.3	-18.56	-9.92	-9.31	<0.001***
IFG - HG	Sen	4.72	2.51	444.4	-2.35	11.79	1.88	0.101
IFG - ITG	Sen	4.11	1.55	297.8	-0.27	8.49	2.66	0.029*

IFG - MTG	Sen	3.39	1.62	266.0	-1.20	7.98	2.09	0.092
IFG - STG	Sen	-0.65	2.01	372.8	-6.32	5.02	-0.32	0.800
HG - ITG	Sen	-0.60	2.38	441.2	-7.33	6.12	-0.25	0.800
HG - MTG	Sen	-1.33	2.41	442.9	-8.12	5.47	-0.55	0.728
HG - STG	Sen	-5.37	2.68	441.3	-12.94	2.20	-2.00	0.092
ITG - MTG	Sen	-0.72	0.92	434.9	-3.32	1.88	-0.78	0.619
ITG - STG	Sen	-4.76	1.49	438.9	-8.97	-0.55	-3.19	0.015*
MTG - STG	Sen	-4.04	1.53	437.3	-8.36	0.28	-2.64	0.029*

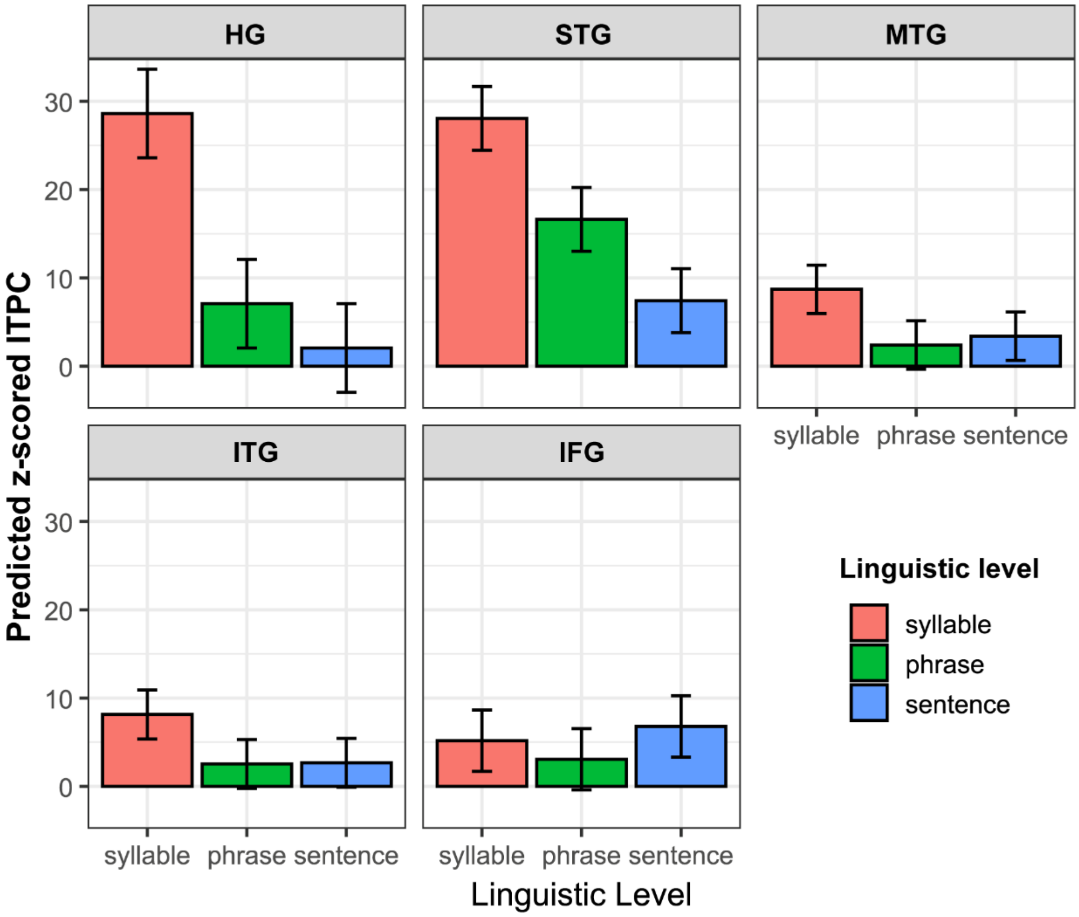

Figure S2 Estimated marginal means of z-scored ITPC across linguistic levels and regions from linear mixed-effects models (region × linguistic level) in the right hemisphere. Error bars indicated 95% confidence intervals (CIs).

Table S5. Localization of contacts with significant high gamma tracking responses to phrases and sentences.

Contact #	Subj.ID	AAL label	MNI coordinates		
			x	y	z
**High-gamma tracking to phrases**					
211	3	Temporal_Sup_L	-56	-30	10
553	8	Frontal_Inf_Orb_L	-49	25	-5
554	8	Frontal_Inf_Orb_L	-53	25	-4
657	9	Heschl_R	41	-17	6
669	9	Temporal_Pole_Sup_R	47	6	-9
745	10	Cingulum_Mid_R	11	37	30
817	10	Cingulum_Mid_R	12	-43	35
1487	18	Insula_L	-33	-21	9
1730	20	Temporal_Sup_L	-55	-16	9
1731	20	Temporal_Sup_L	-59	-17	9
**High-gamma tracking to sentences**					
303	4	Heschl_L	-44	-20	6
305	4	Temporal_Sup_L	-50	-22	6
553	8	Frontal_Inf_Orb_L	-49	25	-5
554	8	Frontal_Inf_Orb_L	-53	25	-4
670	9	Temporal_Pole_Sup_R	51	6	-10
671	9	Temporal_Pole_Sup_R	54	6	-11
1198	14	Rolandic_Oper_L	-37	-26	17
1200	14	Rolandic_Oper_L	-44	-26	19
1201	14	Rolandic_Oper_L	-47	-26	20
1429	17	Heschl_L	-31	-25	7
1430	17	Heschl_L	-34	-26	7
1457	17	Rolandic_Oper_L	-42	-18	15
1615	19	Temporal_Inf_R	42	-42	-14
1616	19	Temporal_Inf_R	45	-44	-14
1681	20	Temporal_Mid_L	-49	-1	-15
1708	20	Temporal_Inf_L	-35	-34	-14
1730	20	Temporal_Sup_L	-55	-16	9
1731	20	Temporal_Sup_L	-59	-17	9

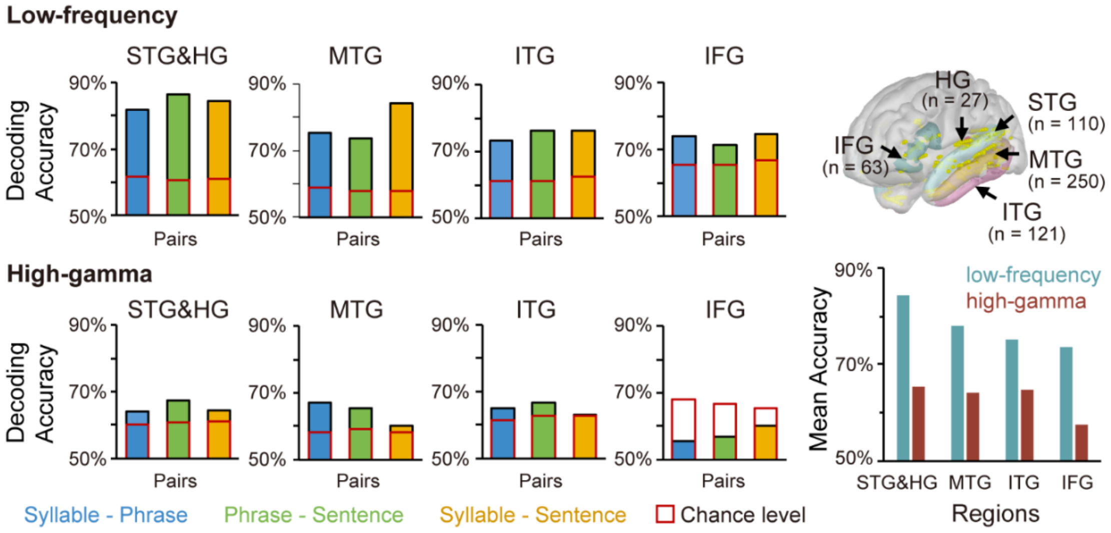

Figure S3. Neural decoding performance using low-frequency and high-gamma activities in the left fronto-temporal network. We included all the recorded contacts localized in the left HG (n = 27), STG (n = 110), MTG (n = 250), ITG (n = 121), and IFG (n = 63). Due to the limited number of contacts localized in the left HG, we merged the data sets of HG and STG together for decoding linguistic structures. The Support Vector Machine (SVM) was employed with a 5-fold cross-validation method, where 4 folds were used for training the model and 1 fold for testing. Additionally, we assessed the chance level by randomizing the data labels. The SVM classifier was repeated for 100 times, and the average decoding accuracy was calculated. This classification approach was applied to the conditions: (1) syllables versus phrases, (2) phrases verses sentences, and (3) syllables versus sentences. The mean decoding accuracy across all pairs showed higher decoding accuracy using low-frequency data compared to high-gamma data. It should be noted that this decoding approach was influenced by the distribution of recorded contacts in different brain regions, which was not randomly sampled, but rather determined by the clinical conditions of each subject's implantation procedure. Thus, care should be taken in interpreting this part of results.

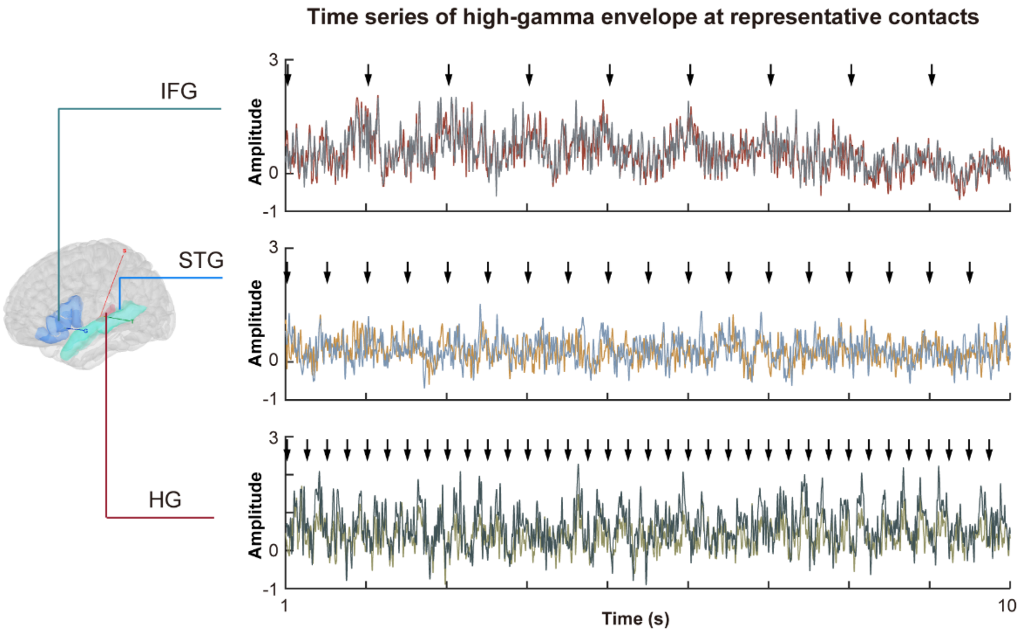

Figure S4. Averaged time courses of high-gamma envelope responses across trials. Sample contacts were located in the left fronto-temporal regions, including the inferior frontal gyrus (IFG; MNI: [−49, 25, −5], [−53, 25, −4]), superior temporal gyrus (STG; MNI: [−55, −16, 9], [−59, −17, 9]), and Heschl's gyrus (HG; MNI: [−39, −17, 6], [−39, −18, 10]). Black arrows indicate the onsets of syllables, phrases, or sentences, depending on the condition.

Table S6. Percentage of responsive sEEG contacts in fronto-temporal regions, along with the number of subjects contributing to each response pattern. The notation "s#" under the contact count and percentage indicates the number of unique subjects who contributed contacts to that category**.** HG, Heschl's gyrus; STG, superior temporal gyrus; MTG, middle temporal gyrus; ITG, inferior temporal gyrus; IFG, inferior frontal gyrus.

Brain region	Contact number (s#)	Syllable 1 Hz (s#)	Syllable 2 Hz (s#)	Syllable 4 Hz (s#)	Phrase 1 Hz (s#)	Phrase 2 Hz (s#)	Phrase 4 Hz (s#)	Sentence 1 Hz (s#)	Sentence 2 Hz (s#)	Sentence 4 Hz (s#)
**Left**										
HG	23	9%	17%	96%		26%	91%	13%	39%	100%
	(9)	(2)	(4)	(9)	0	(4)	(9)	(3)	(6)	(9)
STG	90	0	6%	84%		44%	84%	31%	52%	86%
	(9)		(3)	(8)	0	(8)	(8)	(7)	(8)	(8)
MTG	187	2%		32%		13%	37%	6%	12%	44%
	(13)	(1)	0	(9)	0	(5)	(10)	(6)	(5)	(8)
ITG	68			16%		3%	25%	3%	4%	24%
	(10)	0	0	(4)	0	(2)	(7)	(1)	(2)	(6)
IFG	40			28%		5%	10%	18%	18%	23%
	(6)	0	0	(2)	0	(2)	(2)	(2)	(2)	(2)
**Right**										
HG	4			100%		50%	50%	0	75%	100%
	(2)	0	0	(2)	0	(1)	(1)		(2)	(2)
STG	20		20%	85%		75%	65%	50%	55%	80%
	(3)	0	(1)	(3)	0	(3)	(3)	(2)	(3)	(3)
MTG	63			33%		11%	22%	17%	8%	43%
	(7)	0	0	(7)	0	(2)	(5)	(2)	(1)	(6)
ITG	53			47%	0	0	28%	2%	0	53%
	(5)	0	0	(4)			(3)	(1)		(4)
IFG	23		0	22%	0	4%	26%	39%	9%	9%
	(2)	0		(1)		(1)	(2)	(1)	(1)	(1)
